# Myoepithelioma of the Palatal Minor Salivary Gland: A Case Report

**DOI:** 10.7759/cureus.56305

**Published:** 2024-03-17

**Authors:** Shrishty Bhardwaj, Murugesan Krishnan, Santhosh Kumar M P, Senthil Murugan P, Gheena S

**Affiliations:** 1 Oral and Maxillofacial Surgery, Saveetha Dental College and Hospitals, Saveetha Institute of Medical and Technical Sciences, Saveetha University, Chennai, IND; 2 Oral and Maxillofacial Pathology and Microbiology, Saveetha Dental College and Hospitals, Saveetha Institute of Medical and Technical Sciences, Saveetha University, Chennai, IND

**Keywords:** hard palate, surgical excision, innovative technique, pleomorphic adenoma, minor salivary gland tumor, salivary gland disorders, myoepithelioma

## Abstract

Myoepithelioma is an uncommon benign tumor of the orofacial region arising from the salivary glands. These tumors are composed of specifically myoepithelial cells lacking ductal differentiation and were initially considered as a type of pleomorphic adenoma. Though they commonly arise from the parotid gland, there are a few cases that emerge from the minor salivary glands of the palate and oral cavity. Myoepitheliomas resemble many other tumors arising from the palate including pleomorphic adenoma. This report depicts a case of myoepithelioma of the minor salivary gland of the palate in a 23-year-old patient and the successful management of the lesion.

## Introduction

Myoepithelioma is an uncommon benign tumor of the orofacial region. It comprises specifically myoepithelial cells lacking ductal differentiation [1]. They were initially regarded as a pleomorphic adenoma variant, but they gained recognition as a distinct clinical entity [2], following their first description by Sheldon et al. in 1943 [3]. The main reason to separately classify the lesion was the histological appearance of solitary myoepithelial cells. The parotid gland is the primary site of incidence, however, sporadic instances arise in the minor salivary glands of the oral cavity and palate. Myoepitheliomas in minor salivary glands of the oral cavity occur predominantly in the palate [4]. 

On clinical examination, the lesion typically shows no symptoms and may gradually grow in size over several months or years [5]. These tumors present a spectrum of histomorphological patterns, immune antigenic expressions, and clinical behaviors, prompting the need for further exploration and study. Myoepitheliomas exhibit asymptomatic, slowly growing submucosal masses, occurring predominantly in the third decades of life, although they can occur from the first to ninth decades of life [4]. It can occur equally in both genders with a slight predilection for females. It can mimic other benign and malignant tumors of the orofacial region [6]. Hence, it has to be carefully distinguished from other salivary gland tumors. This report depicts a case of myoepithelioma in the anterior palate in a 23-year-old female patient.

## Case presentation

**Figure 1:**
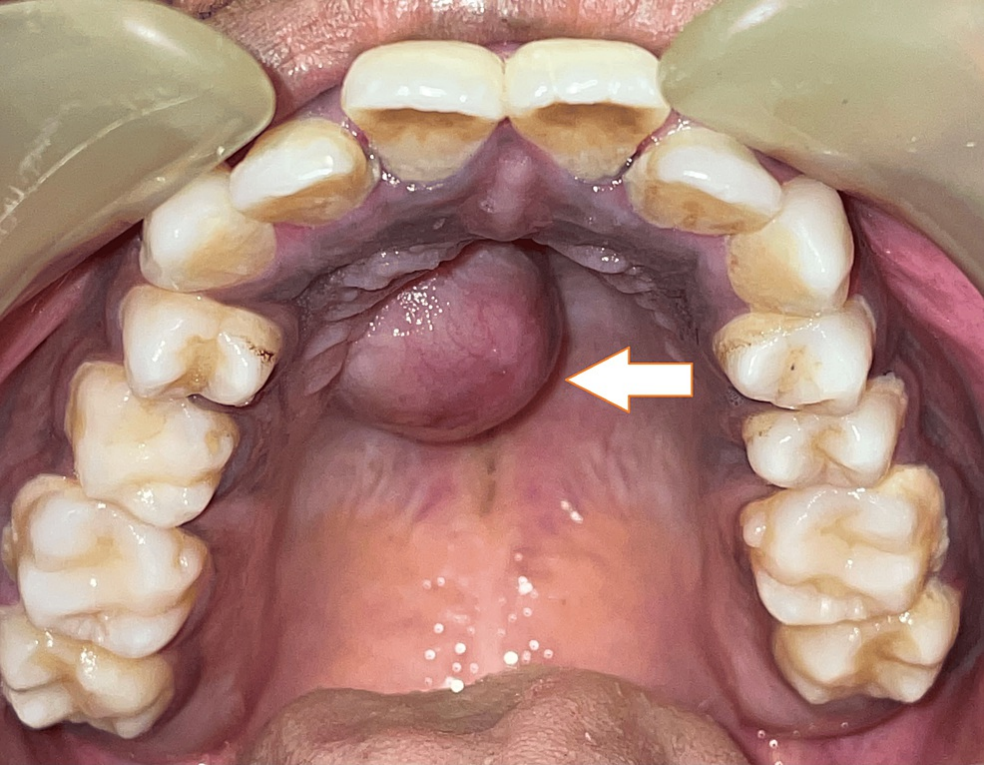
Intraoral picture depicting the lesion (arrow)

**Figure 2:**
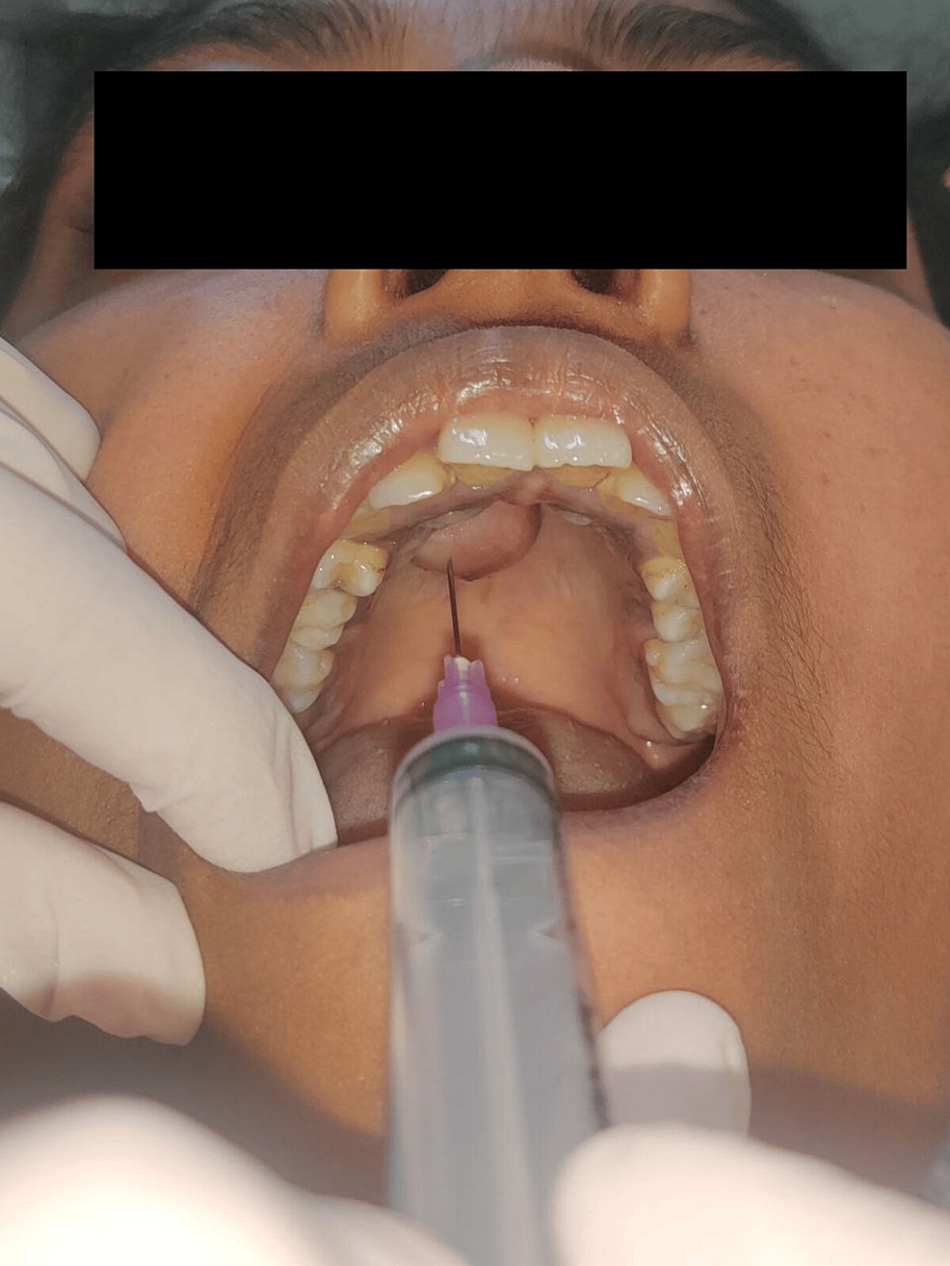
Aspiration of the lesion

A 23-year-old female presented with an asymptomatic, gradually growing mass in the palatal region for the past five months. The patient denied experiencing dysphagia, voice alterations, appetite and weight loss, or fever. The patient's medical history yielded no pertinent information regarding the current condition and negative for habit history. On clinical examination, a 3 x 3 cm, non-tender, non-pulsatile, pinkish round mass extending from the midline to the right side of the anterior hard palate region (extending from 11 to 14 region) was found (Figure 1). The mass exhibited no ulceration or erosion of the overlying mucosa, and there was an absence of cervical lymphadenopathy. The swelling was soft in consistency and fine needle aspiration was done to rule out cystic lesion, which yielded negative results (Figure 2). 

**Figure 3:**
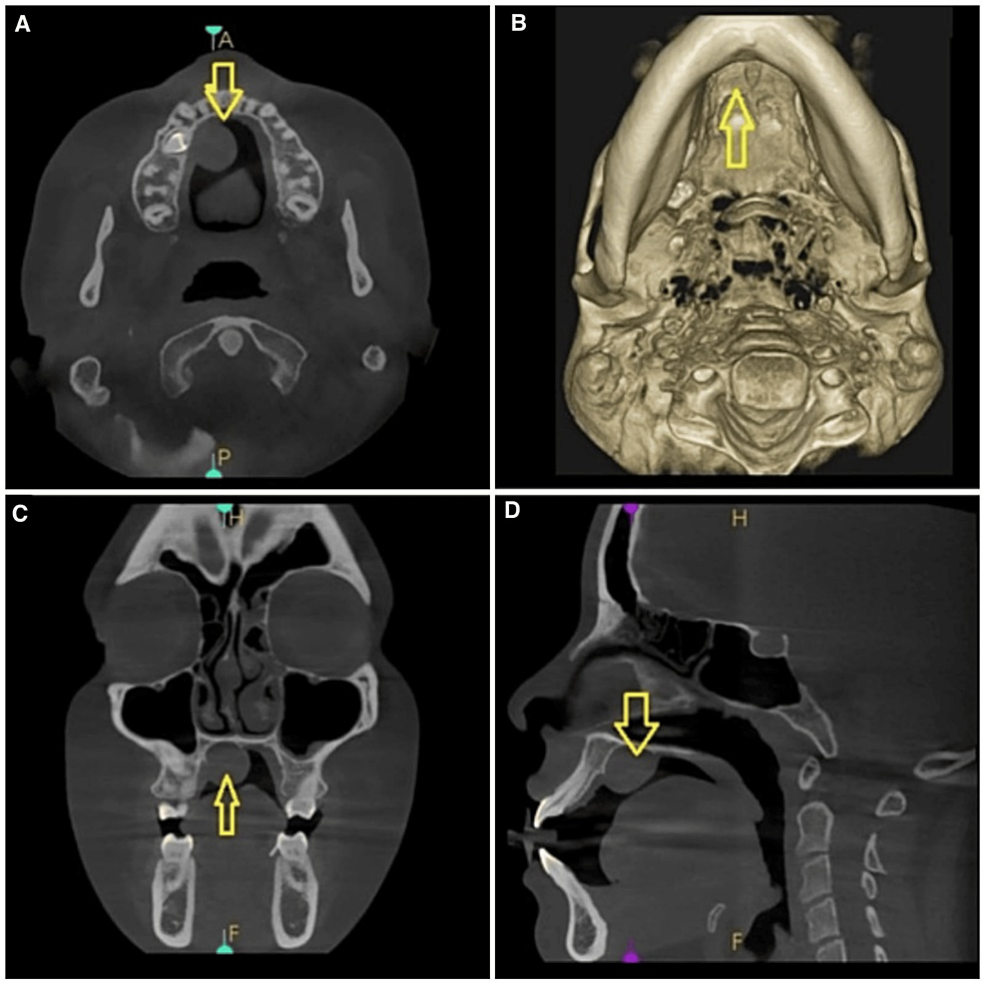
Cone-beam computed tomography sections depicting the lesion (arrow) A: axial section; B: three-dimensional contrast section; C: coronal section; D: sagittal section

Cone-beam computed tomography imaging showed a distinct soft tissue mass emerging from the soft palate, with dimensions of 15 x 14 x 18 mm extending from 11 to 14 region, crossing the midline with no erosion of bony cortices (Figure 3). This mass did not lead to any compromise in the pharyngeal airways. It abutted the hard palate without evident involvement of the hard palate itself. There was an absence of cystic components, calcification, or fat deposits in the mass. This mass did not lead to any compromise in the nasopharyngeal and oropharyngeal airway. Based on the information obtained, the lesion was provisionally diagnosed as pleomorphic adenoma or myoepithelioma arising from minor salivary glands of the palate.

Clinical differential diagnosis

A list of differential diagnoses can be associated with this slow-growing, painless, non-tender, firm, non-ulcerated, sessile mass in the palate. The absence of any aspirate, active discharge, or cystic content rules out palatal abscess or fissural cyst. The patient had no tenderness on percussion in any associated teeth, which also ruled out periapical lesions [7]. A non-fluctuant characteristic of the lesion rules out vascular lesions [8]. The palate consists of minor salivary glands and various lesions can be associated with it including benign lesions like pleomorphic adenoma, myoepithelioma, and basal adenoma [9] and malignant lesions like mucoepidermoid carcinoma, and adenoid cystic carcinoma [10]. Leiomyoma and granular cell myoblastoma can present with similar clinical characteristics, although the palate is a very uncommon site of occurrence for them [11].

Based on the patient's clinical characteristics, the frequency of previously reported lesions, and demographic information, the clinical differential diagnosis for salivary gland tumors was pleomorphic adenoma, basal cell adenoma, myoepithelioma, low-grade mucoepidermoid carcinoma, and polymorphous low-grade adenocarcinoma [12]. Other lesions that were suggested were lipoma, neurofibroma, neurilemmoma, leiomyoma, granular cell myoblastoma, and benign fibrous histiocytoma [13]. However, histological analysis using techniques like fine needle aspiration cytology or incisional/excisional biopsy is the only way to confirm the diagnosis of any soft tissue tumor [14].

**Figure 4:**
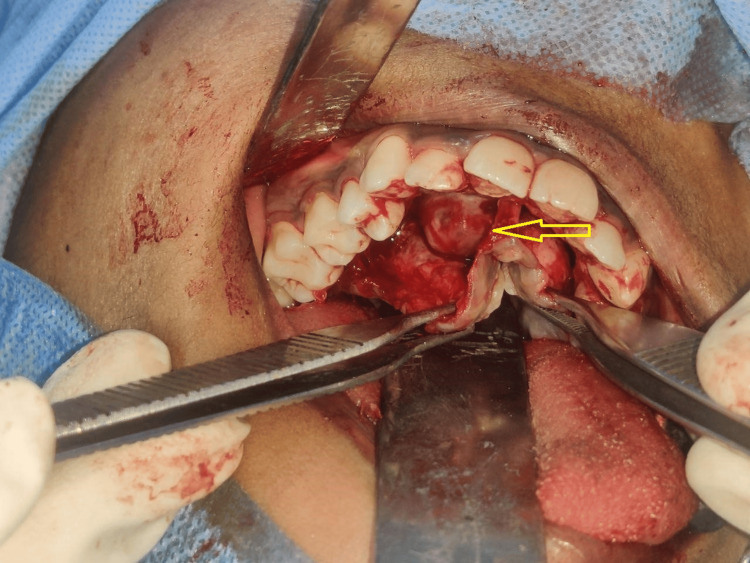
Flap raised and lesion exposed (arrow)

**Figure 5:**
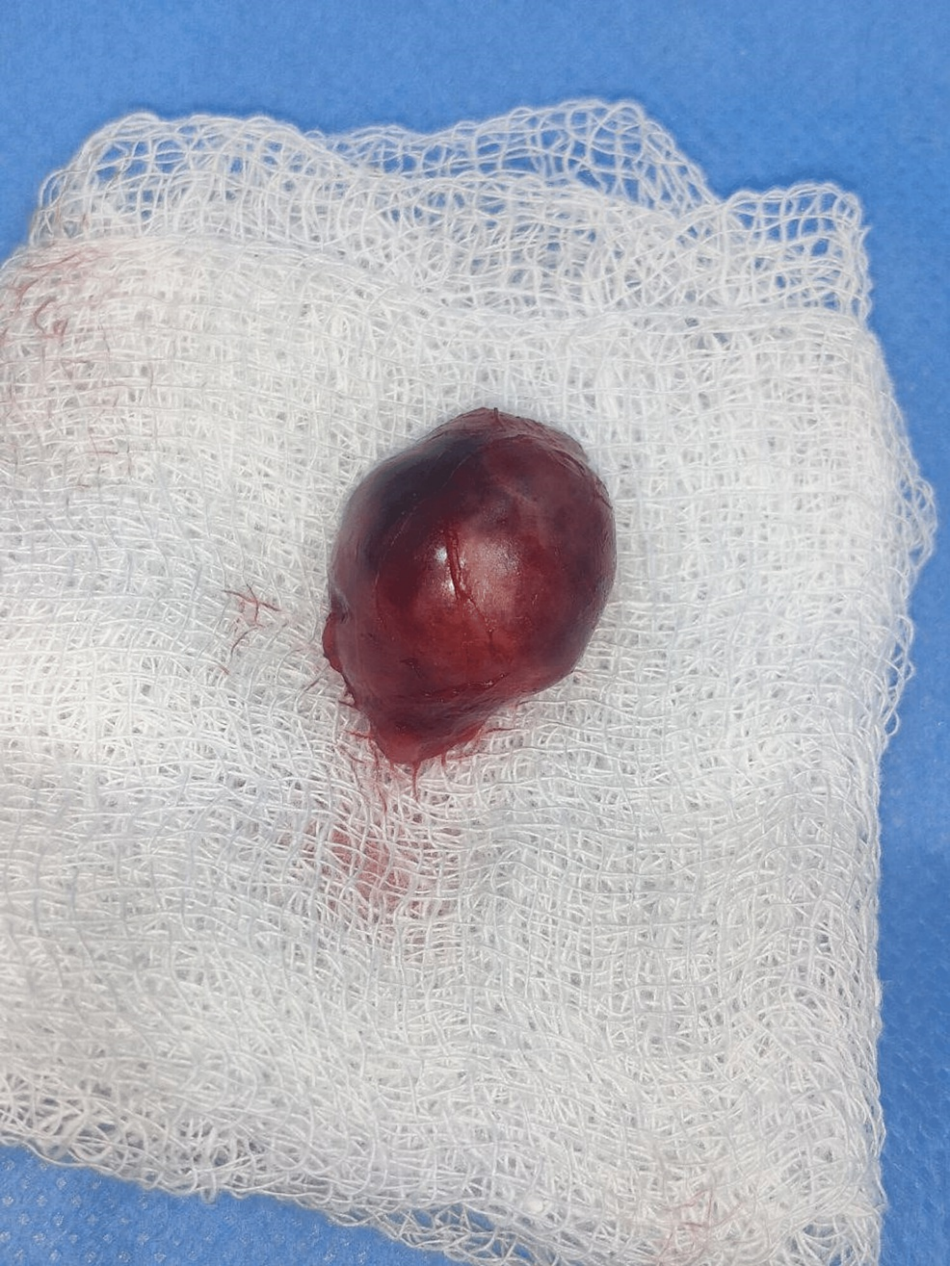
Lesion removed in toto

**Figure 6:**
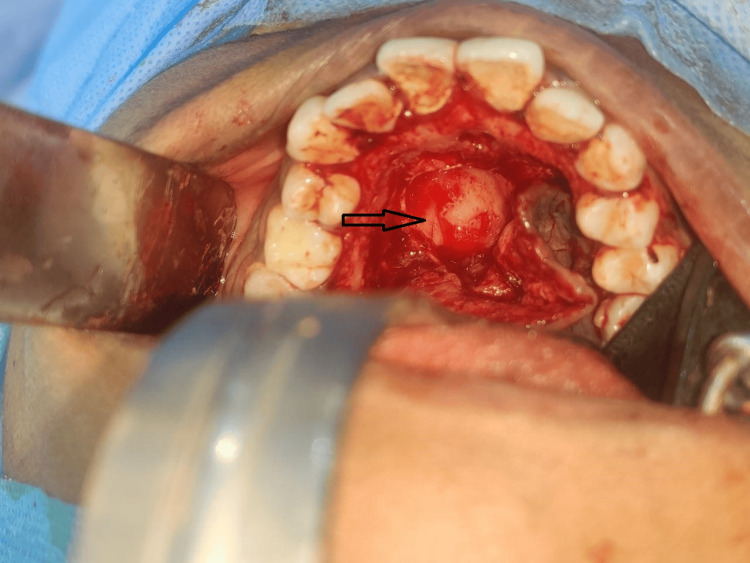
Surgical defect after excision of the lesion showing mild erosion of the palatal bone (arrow)

A conservative treatment plan was formulated considering the patient’s age and the lesion's clinical presentation. Subsequently, a total excision of the lesion was planned and performed under general anesthesia. A crevicular incision was placed, the envelope palatal flap raised, and dissection revealed the tumor presenting as a firm, well-circumscribed, encapsulated lesion (Figure 4). The lesion was excised with adequate safety margins and removed in toto through curettage of the periosteum and mucosa (Figure 5). There was evident erosion of the palatal bone observed after excision of the tumor (Figure 6). Hemostasis was achieved by compression of the wound. The surgical defect site was packed with Bactigras dressing and closed primarily by palatal flap with interrupted sutures. The excised specimen was sent for histopathological examination.

**Figure 7:**
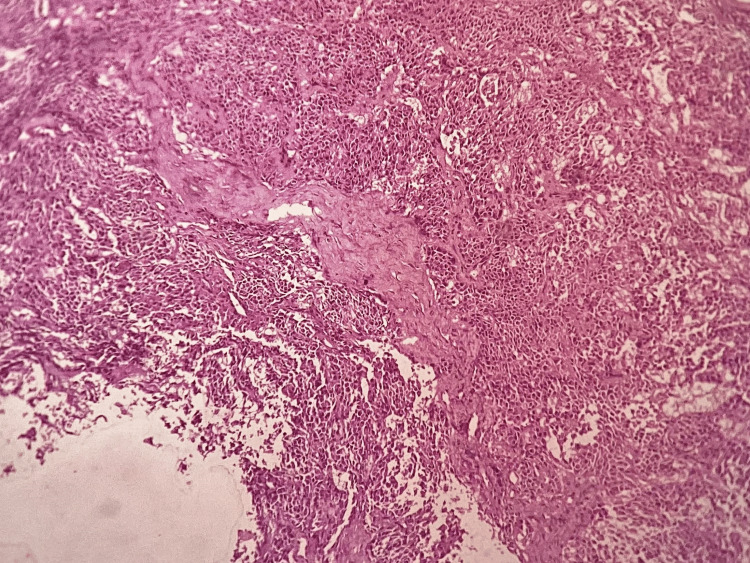
Histopathological picture of the lesion displaying formations of clusters and cords, and sheets comprising large uniform cells exhibiting plasmacytoid characteristics (hematoxylin and eosin stain, 100x magnification)

**Figure 8:**
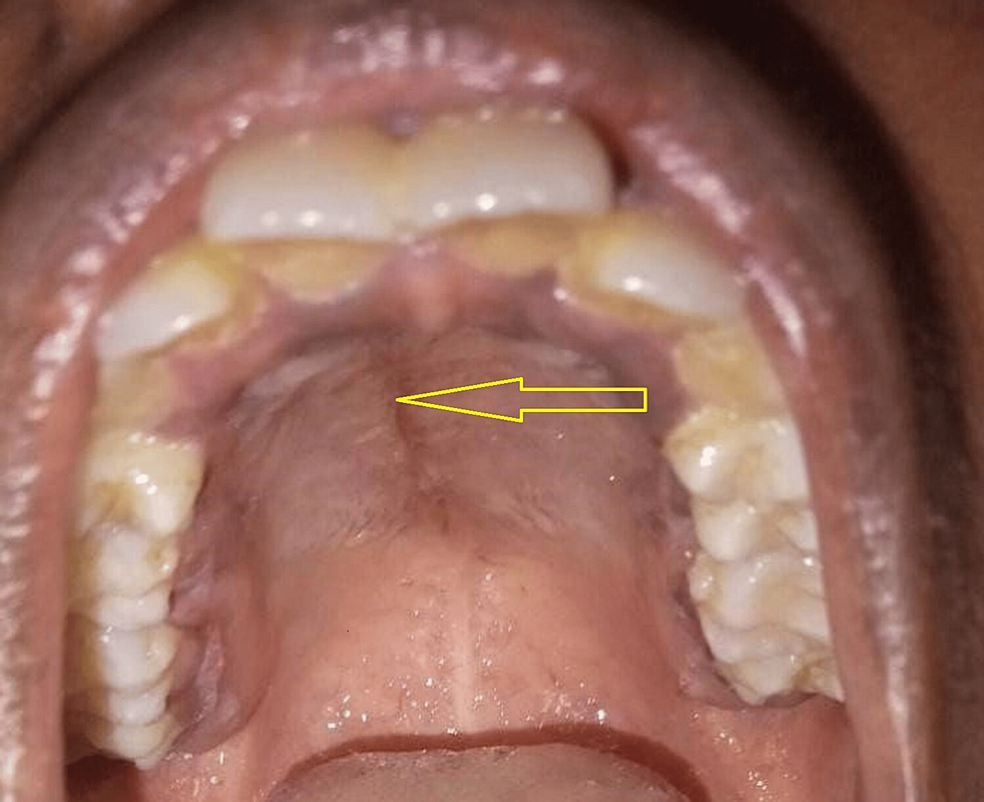
Ten-month postoperative image depicting satisfactory wound healing (arrow)

Under microscopic examination, the tissue exhibited formations of clusters and cords, and sheets comprising large uniform cells showing plasmacytoid characteristics. These cells featured ovoid or round eccentric nuclei. There was also the presence of clear eosinophilic cytoplasm. Notably, an abundant myxoid stroma was observed between these cellular groups. Surrounding the mass was a delicate, fibrous capsule, with small salivary gland acini located outside of it. Acinar and ductal differentiation was notably absent and there were no signs of malignancy. Mitotic figures, interstitial hemorrhage, necrosis, or infiltration into adjacent tissues were absent. The histopathological report was indicative of myoepithelioma (plasmacytoid variant) which is a benign salivary gland neoplastic lesion (Figure 7). There were no signs of recurrence of the mass during the 10-month follow-up period (Figure 8).

## Discussion

Myoepithelioma usually manifests in tissues with secretory functions, including minor and major salivary glands, lacrimal glands, sweat glands, prostate, nasopharynx, breast, and lungs. Predominantly, myoepitheliomas occur in the parotid gland (40%) and minor salivary glands (21%). Literature reports that 40% of cases occur in the soft palate [6]. Minor gland occurrences are slightly more in younger individuals compared to other sites [15], with a similar occurrence seen in this patient.

Myoepithelioma has a peak occurrence in the third decades of life, with a female-to-male ratio of 1.7:1, as observed in reported cases [4], with a similar pattern seen in this patient. Distinguishing myoepithelioma of the soft and hard palate from other palatal tumors, such as neurinomas, pleomorphic adenoma, malignant tumors, hemangiomas, solitary fibrous tumors, metastatic tumors, and other inflammatory diseases, is essential [1]. Due to shared clinical and radiological features among these lesions, biopsy becomes imperative for confirming the diagnosis of myoepithelioma, especially given the challenging differentiation from salivary gland tumors like pleomorphic adenoma.

In this case, an excisional biopsy was conducted, aligning with the optimal approach considering the patient’s age and post-operative rehabilitation. The wide variety of differential diagnoses associated with the lesion, in this case, was a challenging experience. Clinically and demographically, a diagnosis of benign neoplasm, like pleomorphic adenoma, or aggressive lesions like adenoid cystic carcinoma and mucoepidermoid carcinoma can be given. Generally, myoepithelioma occurs in the soft palate [12], making the appearance in this case, which is the hard palate, a confusing characteristic. Intraoperatively, erosion of palatal bone was a very crucial observation, as it was not evident radiographically and is not associated with benign lesions like myoepithelioma. After excision, the lesion was encapsulated, and this is more commonly observed in association with major salivary glands [16]. 

Histopathology reports confirm this lesion to be originating from myoepithelial cells, thereby narrowing down the differential diagnoses to pleomorphic adenoma, myoepithelioma, myoepithelial carcinoma. Pleomorphic adenoma can be confirmed by the presence of chondromyxoid stroma, and more than 5% of cells should have ductal proliferation and eosinophilic coagulum, which was not observed in this lesion. Loss of any cellular atypia and the presence of a fibrous capsule also rule out myoepithelial carcinoma [15]. 

Myoepitheliomas can be viewed as a variant of pleomorphic adenoma, wherein glandular-ductal differentiation is either absent or virtually so. Consequently, the architectural patterns and cellular differentiation in myoepitheliomas closely resemble the non-luminal portions of pleomorphic adenomas [2]. Macroscopically, myoepitheliomas present as well-circumscribed tumors with a glistening yellow-brown, smooth surface, devoid of soft tissue or adjacent bone tissue involvement. Microscopically it manifests in four forms: spindle-cell, epithelioid, plasmacytoid (or hyaline), and clear-cell types. These subtypes may appear individually or in a combined form [16]. Immunohistochemical markers used for myoepithelioma are calponin, actin, P63, pan CK, and CK7. 

The surgical approach for myoepithelioma involves a thorough excision, ensuring a safety margin of uninvolved tissue [5]. Clinicians need to be vigilant in peripheral detachment during surgery to identify potential extensions or invasive tendencies. In this case, the lesion was effortlessly dissected and removed from adjoining tissue after a superficial incision, as no adhesions were observed. Benign myoepithelioma has a good prognosis, which is similar to the mixed tumors, although in the literature recurrences up to 15-18% are reported [17]. There have been no reports of distant dissemination of benign myoepithelioma in the literature [18]. No specific cell type or growth pattern has demonstrated a notable impact on the prognosis of myoepithelioma. However, incomplete tumor resection appears to be associated with a higher recurrence rate in some of the cases [19,20].

This case was a good experience in understanding the plethora of differential diagnoses and the variety of clinical manifestations of palatal minor salivary gland tumors. A conservative approach was made in this case, however intraoperative evidence of bone erosion was misguiding towards a malignant lesion. Post-operative 10-month follow-up shows the patient to be disease-free. These factors in any minor salivary gland lesion are very important in planning the surgical approach and follow-up.

## Conclusions

Myoepithelioma of salivary glands may mimic various lesions and must be considered in the differential diagnosis of salivary gland tumors. The diagnosis of uncommon salivary gland tumors, particularly those emerging in atypical locations with unfamiliar morphological patterns, can be difficult. Increased awareness of the occurrence of these rare lesions is crucial for prompt diagnosis. Many of these tumors can be effectively treated through proper excision and incomplete resection may lead to recurrence of these lesions.
